# Maternal self-esteem and postpartum sexual health in Polish women

**DOI:** 10.3389/fpubh.2026.1813933

**Published:** 2026-06-16

**Authors:** Agnieszka Czerwińska-Osipiak, Wiktoria Rozmarynowska, Aleksandra Krawczyk, Anna Weronika Szablewska, Jolanta Olszewska

**Affiliations:** Division of Obstetric and Gynaecological Nursing, Faculty of Health Sciences with the Institute of Maritime and Tropical Medicine, Medical University of Gdańsk, Gdańsk, Poland

**Keywords:** maternal health, self-esteem, sexual activity after childbirth, sexual behavior, sexual health

## Abstract

**Introduction:**

The postpartum period brings profound physical, psychological and social changes that significantly impact women’s sexual health and relationship quality. Despite its importance for maternal wellbeing and family functioning, postpartum sexual health remains inadequately addressed in clinical practice and research. Self-esteem is a fundamental psychological resource often challenged by postpartum body changes and role transitions; however, it has received limited empirical attention regarding its association with sexual behaviors during this significant period. The aim of this study is to identify factors associated with maternal self-esteem among Polish women, and to examine its associations with selected aspects of postpartum sexual functioning and behaviors. These specifically include the conscious decision to delay first intercourse and ongoing sexual activity frequency.

**Materials and methods:**

This cross-sectional study included 350 Polish women within 1–12 months postpartum, who were recruited through online parenting forums. The participants completed the Rosenberg Self-Esteem Scale and a structured questionnaire assessing demographic characteristics, obstetric history and sexual behaviors. Data were analyzed using chi-square tests, the Mann–Whitney U test and the Kruskal-Wallis test with post-hoc and logistic regression analyses.

**Results:**

Self-esteem demonstrated strong associations with perceived childcare competence (*Z* = 6.08, *p* < 0.001), sexual attractiveness (*H* = 57.89, *p* < 0.001), and partner closeness (*τ* = 0.23, *p* < 0.05). Women with higher self-esteem were significantly less likely to consciously postpone first postpartum intercourse (*M* = 30.60 vs. 28.10, *Z* = 4.35, *p* < 0.001) and reported more frequent sexual activity (*H* = 14.09, *p* = 0.029). Independent predictors of conscious delay included perceived delivery difficulty (OR = 1.55), postpartum sexual frequency (OR = 1.14) and need for self-respect (OR = 1.31).

**Conclusion:**

The findings demonstrate that higher maternal self-esteem is associated with less recurrent conscious postponement of first intercourse and greater sexual activity frequency. Integrating support for maternal self-esteem into clinical practice and public health initiatives should be a crucial component of comprehensive postpartum care.

## 1 Introduction

The postpartum period represents an important period for public health intervention, as maternal physical and psychological wellbeing during this time is profoundly associated with not only individual health outcomes, but also family functioning and child development. While considerable attention has been devoted to postpartum physical recovery and mental health screening, sexual health remains a frequently overlooked dimension of comprehensive postpartum care (1). Nonetheless, sexual wellbeing constitutes an integral component of overall quality of life, with documented associations with relationship satisfaction, psychological adjustment and long-term maternal health (2, 3). In recent systematic reviews, it has been highlighted that the timing and quality of sexual resumption after childbirth significantly impact women’s overall postpartum adjustment and family dynamics (4). Understanding factors that shape postpartum sexual health is therefore essential for delivering holistic, woman-centered care during this life stage.

Self-esteem—defined as an individual’s subjective evaluation of his/her own worth and competence—represents a fundamental psychological resource that may be particularly vulnerable during transition to motherhood. The postpartum period brings profound changes to women’s bodies, identities and daily routines, potentially challenging their sense of self-worth and competence (5). Physical changes—including weight retention, breast alterations and perineal trauma—may affect body image and perceived attractiveness. Recent evidence demonstrates that genital perception and body image concerns are significantly associated with postpartum sexual function (6). Meanwhile, the demands of infant care and role adjustment have been linked to maternal self-efficacy and overall quality of life during early motherhood (2). However, self-esteem may also function as a protective factor, acting as a potential buffer against postpartum stressors and facilitating adaptive coping strategies. In our recent research, it has been shown that self-esteem, alongside maternal age and birth experience, plays a significant role in shaping anxiety before first postpartum intercourse (7).

Although factors affecting postpartum sexuality have been identified in numerous studies, including mode of birth, breastfeeding status, fatigue and perineal trauma (8, 9), the role of psychological factors such as self-esteem has received limited empirical attention. Most of the existing research is focused on sexual dysfunction or satisfaction as outcomes, with women’s active decision-making regarding sexual resumption and the psychological factors shaping it, remaining insufficiently examined (10). Recent qualitative work has begun to explore the concept of “feeling ready” to resume sexual activity, highlighting the complex interplay of physical, emotional and relational factors women navigate in the postpartum period (11). Understanding whether and how self-esteem relates to the conscious decision to delay first postpartum intercourse and to ongoing sexual frequency could provide valuable insights for clinical practice. Moreover, research in this area remains particularly sparse in Central and Eastern European contexts, including Poland, where cultural factors and healthcare practices may shape postpartum experiences differently than in widely studied Western populations (12). Identifying modifiable psychological correlates of postpartum sexual health could inform the development of targeted interventions to support women during this transition (13).

Such gaps are identified in the present study through three specific aims. First of all, we sought to recognize significant psychosocial correlates of maternal self-esteem in the postpartum period, including perceived competence in childcare, sexual attractiveness and partner relationship quality. Secondly, we examined associations between self-esteem levels and two selected sexual health behaviors: the conscious decision to postpone first postpartum intercourse and the frequency of ongoing sexual activity after childbirth. Moreover, it was explored whether breastfeeding status is associated with these patterns. Finally, we investigated additional determinants of the decision to delay sexual resumption beyond self-esteem, including birth-related factors and previous sexual patterns. We hypothesized that higher self-esteem would be associated with less frequent conscious postponement of sexual activity and greater sexual frequency, and that self-esteem would correlate positively with perceived competence, attractiveness and relationship closeness.

## 2 Materials and methods

### 2.1 Study design and participants

We conducted a cross-sectional observational study examining self-esteem and sexual health outcomes among Polish women during their first year postpartum. The research aim was to identify relationships between maternal self-esteem levels, as measured by the Rosenberg Self-Esteem Scale, and patterns of sexual activity resumption following childbirth.

Recruitment occurred through social media platforms, specifically targeting Polish parenting forums and maternal support groups with a combined membership of approximately 15,000 individuals. To reduce selection bias, we excluded private or geographically restricted groups. Women were eligible if they: had delivered within the previous 12 months, maintained a stable partner relationship throughout pregnancy and postpartum, and provided informed consent. Using Computer-Assisted Web Interview (CAWI) technology, we collected responses from 350 eligible participants.

Based on *a priori* statistical power calculations conducted in G*Power, it was determined that 176 participants would provide adequate power (80%) to detect moderate correlations (*r* = 0.3) at *α* = 0.05. This anticipated effect size reflects published correlations between psychological constructs and postpartum sexual functioning (*r* = 0.25–0.35) (14). Effect size conventions followed Cohen’s guidelines for behavioral research (15). Our achieved sample size (*N* = 350) exceeded this requirement, yielding 97.7% power.

The study protocol received approval from the Independent Bioethics Committee for Scientific Research at the Medical University of Gdańsk (approval number: NKBBN/1017/2021-2022) and followed both STROBE reporting guidelines (16) and the 1964 Declaration of Helsinki. Quality control measures included attention check items and automated detection of duplicate responses based on IP addresses.

### 2.2 Measures and variables

Demographic and clinical characteristics: A structured questionnaire, collaboratively developed by experienced midwifery clinicians, captured relevant background information across 34 items. These addressed: demographic factors (maternal age, educational attainment, residential setting, relationship status), reproductive history (number of prior births, postpartum interval, birth method and subjective difficulty, partner attendance at birth), and current circumstances (infant feeding method, perceived competence in infant care, relationship satisfaction).

Self-esteem assessment: We administered the Polish-language version of the Rosenberg Self-Esteem Scale (RSES), a widely validated 10-item instrument. Respondents rate agreement with statements on a four-point scale, generating total scores from 10 to 40, with higher values reflecting more positive self-regard. Polish population norms for women indicate a mean of 29.19 (*SD* = 4.28) (17). The scale demonstrated strong reliability in our sample (Cronbach’s *α* = 0.92). Although scores were analyzed continuously in the majority of tests, we also categorized them into tertiles (low/moderate/high self-esteem) for selected analyses examining associations with categorical variables.

In the present study, selected aspects of sexual health are operationalized through several behavioral and psychosocial dimensions—specifically the autonomous decision regarding timing of sexual resumption, coital frequency, perceived sexual attractiveness and pre-intercourse anxiety. These indicators come from the WHO (2017) multidimensional framework on sexual health, but they capture only selected dimensions rather than providing a comprehensive assessment of sexual health or clinical sexual function (18).

Sexual behaviors and related outcomes: Our primary outcomes included whether women deliberately delayed resuming intercourse (dichotomous: yes/no); current coital frequency (seven ordered categories from ‘daily’ to ‘not yet resumed’); presence and intensity of anxiety preceding first postpartum intercourse (dichotomous presence, then five-point intensity rating); subjective sexual attractiveness (three categories: yes/no/uncertain); and perceived emotional closeness with partner (dichotomous: yes/no).

Conscious postponement of first postpartum intercourse was assessed using a single author-developed item: “Did you consciously delay first postpartum sexual intercourse with your partner?”, with response options of ‘yes’ or ‘no’. Among women who reported a deliberate delay (*N* = 155), a subsequent multi-choice item was used to assess the reasons for postponement. The response options included: fatigue, perceived lack of sexual attractiveness, body shape changes, reluctance for intimacy, lack of time, perineal wound pain, pain following cesarean section, and shame (multiple responses permitted). The item was not previously validated as a standalone instrument, which is acknowledged as a limitation.

### 2.3 Statistical analysis

All statistical calculations were performed using IBM SPSS Statistics version 23 and Microsoft Excel 2016. Descriptive statistics for sample characteristics included frequencies and percentages for categorical variables as well as means with standard deviations for continuous variables.

Our analytical strategy then proceeded through several stages. We first employed chi-square tests to examine associations between self-esteem categories (low/moderate/high) and significant variables including childcare competence, sexual attractiveness and partner closeness. The Mann–Whitney U test was used to compare self-esteem scores between women who did and did not consciously postpone first postpartum intercourse. To examine differences in self-esteem across sexual frequency groups, we applied the Kruskal-Wallis test with post-hoc Bonferroni comparisons for multiple group comparisons. Spearman’s rank correlation coefficient was utilized to assess relationships between continuous variables and non-parametric distributions.

Finally, logistic regression analysis was conducted to identify independent predictors regarding conscious postponement of first intercourse. The model included perceived birthing difficulty, frequency of intercourse after previous births and the need for self-respect as predictor variables. Self-esteem was examined separately via non-parametric tests to establish bivariate associations prior to modeling, allowing us to understand its relationship with sexual behaviors before multivariable modeling. Consequently, global self-esteem was not entered as a predictor in the logistic regression model, which was designed to identify additional independent predictors of conscious postponement operating over and above the self-esteem–delay association documented in the bivariate analyses. Model fit was assessed using the Hosmer-Lemeshow goodness-of-fit test, with pseudo-*R*^2^ values (Cox-Snell and Nagelkerke) reported to indicate the proportion of explained variance.

The significance level was set at *p* ≤ 0.05 for all analyses. For logistic regression, odds ratios (OR) with 95% confidence intervals (CI) were calculated to quantify the strength of associations.

## 3 Results

### 3.1 Sample characteristics

Multiple correspondence analysis generated a Burt table that revealed the following profile of the typical participant based on categories with the highest contribution to variance: a married or cohabiting woman with higher education, aged 25–29 years, residing in a rural area or small town. She was primiparous, having given birth via cesarean section without partner presence, and was currently breastfeeding. The typical participant felt close to her partner and perceived her childcare competence as good, although she maintained average self-esteem levels and experienced anxiety before resuming postpartum sexual activity. Notably, the typical profile included lack of regular sexual contact during pregnancy and perception that healthcare providers inadequately addressed the topic of return to sexual activity. This profile contextualizes sample characteristics rather than proposing a predictive or typological model.

Detailed demographic and final delivery characteristics of the study population are presented in Table 1.

**Table 1:** Study group characteristics.

Characteristics of the study group	No.	%
Respondents	350	100
Age		
≤25	94	26.9
26–34	236	67.4
35–40	20	5.7
Education level		
Lower secondary (e.g., completed junior high school)	2	0.6
Vocational	11	3.1
Secondary	117	33.4
Higher (university degree)	220	62.9
How much time has passed since your last childbirth?		
About 1 month	1	0.3
Up to 2 months	15	4.3
Up to 3 months	77	22.0
Up to 4 months	47	13.4
Up to 5 months	4	1.1
Up to 6 months	51	14.6
Up to 7 months	71	20.3
Up to 8 months	61	17.4
Up to 9 months	19	5.4
Up to 11 months	1	0.3
Up to 12 months	3	0.9
How would you rate your last childbirth?		
1 – very easy	46	13.1
2 – easy	74	21.2
3 – neither difficult nor easy	105	30.0
4 – difficult	79	22.6
5 – very difficult	46	13.1
How often do you have sexual intercourse with your partner (after childbirth)?		
Daily	8	2.3
2–3 times a week	80	22.9
Once a week	91	26.0
Once every 2 weeks	60	17.1
Once a month	42	12.0
Less often than once a month	33	9.4
I have not had intercourse with my partner yet	36	10.3
Did you consciously postpone the first sexual intercourse after childbirth?		
Yes	155	44.3
No	195	55.7

The survey concluded with administration of the Morris Rosenberg Self-Esteem Scale in its Polish adaptation. Respondents were asked to indicate their level of agreement or disagreement with 10 diagnostic statements using a four-point rating scale presented in Table 2.

**Table 2:** Results of SES-PL scale questionnaire.

Descriptive statistics	*N*	Min	Max	*M*	*SD*
I consider myself to be at least as worthy as others	350	1	4	1.66	0.65
I feel that I have a number of good qualities	350	1	3	1.70	0.60
Generally speaking, I tend to think that things are not going well for me	350	1	4	3.14	0.63
I am able to do things as well as most other people	350	1	4	1.83	0.62
I feel I do not have much of which to be proud	350	1	4	2.99	0.72
I assume a positive attitude towards myself	350	1	4	2.05	0.67
On the whole, I am satisfied with myself	350	1	4	2.08	0.68
I wish I could have more respect for myself	350	1	4	2.49	0.88
All in all, I am inclined to think that I am a failure	350	1	4	2.62	0.87
I certainly feel useless at times	350	1	4	2.59	0.88

### 3.2 Correlates of self-esteem

Correlation and comparative analyses were conducted to examine factors associated with maternal self-esteem in the postpartum period. Self-esteem showed significant associations with several important variables related to maternal adjustment, sexual wellbeing and relationship quality.

*Main factors*: Three primary factors emerged as strongly associated with self-esteem levels. Childcare competence exhibited a significant relationship, with women who rated their ability to care for their infant as “very good” exhibiting markedly higher self-esteem (*M* = 31.76, *SD* = 5.71) compared to those rating their competence as “good” (*M* = 28.05, *SD* = 4.83; *Z* = 6.08, *p* < 0.001). Sexual attractiveness was strongly associated with self-esteem (*H*(2) = 57.89, *p* < 0.001). The women feeling sexually attractive reported the highest self-esteem levels (*M* = 32.49, *SD* = 4.89), and significantly exceeded those who did not feel attractive (*M* = 27.13, *SD* = 5.54) or were uncertain (*M* = 29.27, *SD* = 4.53). Partner closeness showed a robust positive correlation with self-esteem (Kendall’s *τ* = 0.23, *p* < 0.05; *Z* = 5.07, *p* < 0.001). Women reporting close emotional bonds with their partners were significantly more likely to exhibit high self-esteem (44.30%) compared to those not feeling close to their partner (13.46%; *χ*^2^(2) = 23.48, *p* < 0.001). Detailed results are presented in Supplementary Table S1.

*Secondary factors*: Age showed a significant positive association with self-esteem (Kendall’s *τ* = 0.10, *p* < 0.05; *χ*^2^(6) = 14.78, *p* = 0.022). The older women demonstrated higher self-esteem levels. The proportion of women with high self-esteem increased progressively with age: 29.82% among women ≤24 years, 35.47% in the 25–29 age group, 46.53% in the 30–34 age group, and 70.00% among women ≥35 years. Breastfeeding status showed a significant correlation with self-esteem (*χ*^2^(2) = 6.25, *p* = 0.044), and the breastfeeding women were more likely to report high self-esteem (63.31%) compared to those not breastfeeding (36.69%). Additionally, breastfeeding was associated with sexual behavior, as breastfeeding women were significantly more likely to consciously postpone sexual activity (*χ*^2^(1) = 4.77, *p* = 0.029). Detailed results are presented in Supplementary Table S1.

### 3.3 Self-esteem and sexual health behaviors

Self-esteem demonstrated a significant association with postpartum sexual behaviors, including both the timing of sexual resumption and frequency of sexual activity.

Conscious postponement was treated as a neutral behavioral outcome reflecting women’s autonomous decision-making; it may represent either deliberate, recovery-oriented delay or avoidance related to pain, anxiety or relational factors.

*Higher self-esteem associated with less conscious delay*. The Mann–Whitney U test revealed a significant relationship between self-esteem levels and the decision to postpone first postpartum intercourse (*Z* = 4.35, *p* < 0.001). Women who did not consciously delay sexual resumption demonstrated significantly higher self-esteem (*M* = 30.60, *SD* = 5.51) compared to those who intentionally postponed their first intercourse (*M* = 28.10, *SD* = 5.26). This suggests that women with greater self-confidence and positive self-regard felt more prepared to resume sexual activity without deliberate postponement (Table 3).

**Table 3:** SES vs. conscious delay of first intercourse after childbirth.

SES vs. conscious delay of first intercourse after childbirth	*N*	*M*	*SD*	*Z*	*p*
Yes	155	28.10	5.26	4.35	<0.001
No	195	30.60	5.51		

*Higher self-esteem associated with more frequent intercourse*. Kruskal-Wallis analysis showed a significant association between self-esteem and frequency of postpartum sexual activity (*H*(6) = 14.09, *p* = 0.029). Post-hoc comparisons using the Bonferroni correction revealed that women engaging in daily intercourse reported significantly higher self-esteem (*M* = 33.37, *SD* = 5.12) than those having intercourse less than once per month (*M* = 28.06, *SD* = 4.45). Although differences among intermediate frequency groups were not statistically significant, a general pattern emerged, indicating that higher self-esteem was associated with more frequent sexual activity. Women with the lowest occurrence of sexual activity (less than monthly or no intercourse yet) consistently demonstrated lower self-esteem scores across the sample (Table 4).

**Table 4:** SES vs. sexual activity frequency after childbirth.

SES vs. sexual activity frequency after childbirth	*N*	*M*	*SD*	*H*	*df*	*p*
Daily	8	33.37	5.12	14.09	6	0.029
2–3 times a week	80	31.06	5.68			
Once a week	91	29.31	5.16			
Once every 2 weeks	60	28.33	6.24			
Once a month	42	28.97	5.50			
Less than once a month	33	28.06	4.45			
Not yet resumed	36	29.47	5.12			

### 3.4 Other determinants of delayed resumption

Of the 350 participants, 155 women (44.3%) reported consciously postponing first postpartum intercourse for a range of psychosocial and physical reasons. The most frequently cited factors included fatigue (17.4% of responses), perceived lack of sexual attractiveness (15.9%), body shape changes (14.2%), reluctance for intimacy (14.0%) and lack of time (14.0%). Physical factors, including perineal wound pain (8.3%) and pain following cesarean section (8.0%), were also reported. In order to identify independent predictors of this decision, a logistic regression model was constructed. Based on model comparison analysis, the optimal model included three independent variables and an intercept term.

The model exhibited statistically significant predictive power (likelihood ratio test: *p* < 0.000001), although the overall model fit was modest (Pseudo *R*^2^ = 0.12, Nagelkerke *R*^2^ = 0.20, Cox-Snell *R*^2^ = 0.15). The Hosmer-Lemeshow test indicated good model calibration (*p* = 0.42), suggesting adequate agreement between observed and predicted probabilities.

Three factors emerged as significant predictors of conscious postponement:

Perceived difficulty of last birth (OR = 1.55, 95% CI [1.00, 2.41]): women who perceived their last delivery as more difficult were at a higher odds of consciously postponing first postpartum intercourse.

Frequency of intercourse after previous birth (OR = 1.14, 95% CI [1.03, 1.27]): women who reported more frequent sexual activity after previous births demonstrated greater odds of conscious postponement, suggesting that prior positive postpartum sexual experiences may inform more deliberate decision-making regarding sexual resumption.

Need for self-respect (OR = 1.31, 95% CI [1.07, 1.60]): women with a stronger need for self-respect showed increased odds of postponing sexual activity, indicating that self-protective psychological needs were associated with the timing of sexual resumption.

It is important to clarify why self-esteem—the central construct of this study—was not retained in the final logistic regression model. The analytic strategy was sequential: bivariate associations between self-esteem and conscious postponement were first established using non-parametric tests (Table 3). The logistic regression then identified independent predictors among a broader variable set. When perceived birth difficulty, prior sexual frequency and the need for self-respect were entered, global self-esteem was no longer a significant independent predictor. This suggests that its relationship with conscious postponement is either indirect or partially accounted for by the need-for-self-respect item, which captures a specific motivational dimension of self-regard rather than global self-worth. Therefore, the final model reflects the most parsimonious set of statistically independent predictors in this dataset. The logistic regression model for the variables analyzed in the study group (*n* = 350) is presented in Table 5.

**Table 5:** Logistic regression model for conscious postponement of postpartum sexual activity.

Variable	*β*	*SE*	95% CI *(β)*	Wald *χ*^2^	*p-*value	OR	95% CI (OR)
Intercept	−3.702	0.549	[−4.778, −2.627]	45.54	<0.001	0.025	[0.008, 0.072]
Perceived difficulty of last birth (1–5 scale)	0.215	0.099	[0.022, 0.408]	4.77	0.029	1.24	[1.02, 1.50]
Frequency of intercourse after previous birth	0.376	0.074	[0.232, 0.520]	26.07	<0.001	1.46	[1.26, 1.68]
Need for self-respect (RSES item)	0.535	0.140	[0.261, 0.810]	14.66	<0.001	1.71	[1.30, 2.25]

Variables were entered simultaneously using the enter method. Model fit: Hosmer–Lemeshow *χ*^2^(8) = 8.93, *p* = 0.42; Nagelkerke *R*^2^ = 0.20. OR = odds ratio; CI = confidence interval; SE = standard error.

## 4 Discussion

Our findings reveal that maternal self-esteem in the postpartum period functions as a psychological resource built upon multiple interconnected factors. Women with higher self-esteem consistently showed greater perceived competence in childcare, stronger feelings of sexual attractiveness and closer emotional bonds with their partners, aligning with research showing that self-esteem during the transition to motherhood is closely tied to relationship satisfaction and overall adjustment (19–21). The strong association between childcare competence and self-esteem suggests that maternal self-efficacy serves as an essential component of postpartum self-worth. The connection between perceived sexual attractiveness and self-esteem mirrors findings across diverse populations. Hutchinson and Cassidy reported that body dissatisfaction was linked to significantly lower self-esteem in postpartum women, and Meireles et al. similarly found poorer body appreciation and lower self-esteem postpartum compared to pregnancy (20, 22). Zayed and El-Hadidy further demonstrated correlations between sexual satisfaction, body perception and self-esteem in a female sample (23). Our findings extend this pattern: women feeling sexually attractive reported markedly higher self-esteem than those not feeling so, underscoring body image as a critical determinant of maternal psychological adjustment (20, 22, 23). Partner closeness further emphasizes that self-esteem exists within a relational context, in which emotional intimacy reinforces positive self-regard. These findings indicate that self-esteem operates as a multidimensional construct, associated with physical self-perception, maternal identity and relationship quality.

The relationship between self-esteem and postpartum sexual behavior appears bidirectional in existing literature on the subject. Van Scheppingen et al. documented declines in relationship satisfaction and self-esteem during transition to motherhood, while Acele and Karaçam reported that sexual problems in the first postpartum year were associated with psychological distress, including low self-esteem. In our previous study, we found that women with higher self-esteem reported more frequent sexual activity and lower anxiety before resuming intercourse (7, 14, 19). The current findings broaden this pattern, demonstrating that women who did not consciously postpone first postpartum intercourse exhibited higher self-esteem compared to those who deliberately delayed it, and self-esteem showed a dose–response relationship with sexual frequency. This pattern of associations suggests that higher self-esteem may be a marker of greater readiness to resume sexual activity or, alternatively, that positive intimate experiences contribute to higher self-esteem. These cross-sectional findings are consistent with the view that self-esteem and sexual resumption are positively correlated, but they do not permit drawing causal conclusions about the direction of this relationship.

Women who consciously delayed resumption most frequently cited fatigue, perceived lack of sexual attractiveness, body shape changes, reluctance for intimacy and lack of time. A smaller subset also reported physical factors, including perineal wound pain and pain following cesarean section. These reasons collectively reflect the profound life changes accompanying new motherhood. As noted by Martínez-Martínez and colleagues, women require time to accept their new body shape before fully experiencing satisfaction with their sexuality (24). Lower self-esteem during this adjustment may manifest as conscious avoidance of intimacy, representing a protective strategy rather than dysfunction. It is important to note that our single-item measure does not permit a clean distinction between adaptive, recovery-related delay and delay driven by clinically significant fear or pain. In future research, employ multi-dimensional measures should be employed to disentangle such motivations. These findings extend our previous work (7) by demonstrating that self-esteem is associated with actual postpartum sexual behaviors. Self-esteem may serve as a psychological resource correlated with women navigating postpartum sexuality and having greater confidence as well as agency, which resonates with qualitative research on “feeling ready” for sexual resumption (11, 25, 26).

While self-esteem emerged as important, logistic regression revealed that conscious postponement operates through multiple independent pathways. Perceived birthing difficulty and need for self-respect increased postponement odds, while higher frequency of intercourse after previous births reduced the likelihood of delay. Notably, self-esteem was not retained in this final model. An important methodological consideration warrants clarification regarding the distinction between global self-esteem and need for self-respect. Although the need for self-respect is assessed as one item within the Rosenberg Self-Esteem Scale, its emergence as an independent predictor, when global self-esteem was not retained, reflects a conceptually meaningful distinction. Global self-esteem represents a stable trait reflecting overall self-worth across domains. The specific item “I wish I could have more respect for myself”, by contrast, captures a normative-motivational dimension, a value-based decision criterion rather than a current self-evaluation (27). Nevertheless, this interpretation should remain cautious, as the item derives from the same self-esteem scale and its independence from global self-esteem requires replication using separate, validated measures of self-respect and self-esteem in future studies. Women endorsing this need may consciously prioritize physical and psychological recovery, viewing delayed resumption as self-protection rather than failure of confidence. This aligns with research emphasizing that women’s choices reflect complex negotiations between physical readiness, emotional needs, partner expectations and personal values (11). Thus, while high global self-esteem may facilitate earlier activity through reduced body concerns, the need for self-respect operates through deliberate prioritization of personal needs over external pressures, regardless of baseline self-worth. This underscores that conscious postponement may represent healthy self-care rather than dysfunction.

Women perceiving the birth as more difficult may delay sexual activity due to physical healing concerns, pain fears or traumatic experiences, operating independently of global self-esteem, which is consistent with research on the impact of birth trauma (9, 28, 29). The counterintuitive positive association between previous postpartum sexual frequency and current delay may reflect greater sexual self-awareness among multiparous women. The inclusion of need for self-respect suggests some women consciously prioritize their needs over partner expectations—reflecting healthy self-advocacy rather than low self-worth.

### 4.1 Public health and clinical implications

The obtained findings have direct implications for care delivery. Routine postpartum check-ups should incorporate non-judgmental discussions about sexual wellbeing and self-esteem. Rather than simply asking “Have you resumed sexual activity?”, providers could explore readiness, body image concerns and relationship dynamics through questions such as: “How do you feel about your body since giving birth?” or “Do you feel confident caring for your baby?”, to identify women needing additional support.

The critical role of healthcare providers cannot be overstated. As emphasized by Acele and Karaçam, professionals should focus more attention on women’s sexual health throughout the perinatal continuum (14). Lack of knowledge and adequate preparation for childbirth and postpartum changes can generate anxiety and elevated stress levels, which—in turn—may negatively impact both self-esteem and sexual function. Our findings support this concern: 88.3% of women in our sample reported that healthcare providers did not adequately address the topic of postpartum sexual activity resumption. This suggests that current healthcare practices are failing to proactively address a common and important aspect of postpartum adjustment, leaving women to navigate these concerns largely on their own or through potentially unreliable sources.

Healthcare providers—particularly midwives who often maintain continuous relationships with postpartum women—are uniquely positioned to address these issues but require adequate training in sexual health communication (10, 30).

Postpartum support programs—parenting classes, peer groups, online communities—should actively build maternal competence as well as positive body image, as these bolster self-esteem and facilitate healthier sexual adjustment. Interventions validating diverse body changes, normalizing gradual recovery and celebrating maternal capabilities could counter unrealistic expectations (13, 31, 32). Care must be individualized: women experiencing birth trauma require different support (pelvic floor physiotherapy, trauma-informed counseling) than those struggling with body image (peer support, cognitive-behavioral approaches). Given that 44.3% consciously delayed resumption due to modifiable factors, public health initiatives should normalize these experiences while providing practical strategies that would include education about realistic recovery timelines, guidance on intimate communication and reassurance that collaborative delayed resumption generally does not harm relationships (33, 34).

### 4.2 Limitations and future research

This study has several limitations. The cross-sectional design precludes causal inference and limits our ability to determine the direction of the observed associations. It remains possible that higher self-esteem facilitates earlier sexual resumption, satisfying sexual experiences enhances self-esteem, or both are associated with a third, unmeasured factor (e.g., partner support). Longitudinal designs tracking women from pregnancy through postpartum would be necessary to clarify these temporal and potentially bidirectional relationships (26, 35).

The logistic regression model did not include several potentially significant confounders, such as postpartum depressive symptoms, dyspareunia, obstetric complications or a standardized measure of relationship satisfaction. The absence of these variables raises the possibility of unmeasured confounding, and the modest pseudo-*R*^2^ values (Nagelkerke *R*^2^ = 0.20) indicate that the model explains only a limited proportion of the variance in conscious postponement. In future research, more comprehensive multivariable models should be included.

Self-report measures introduce potential social desirability bias, although the anonymous online format may have mitigated this issue. Recruitment through online parenting forums may have introduced selection bias, yielding a sample with higher digital literacy and disproportionately high educational attainment (62.9% held university degrees), which—while reflecting the national trend of increasing educational attainment among Polish women (36)—likely does not represent the broader population of postpartum women in Poland, particularly those with lower socioeconomic status, lower educational levels or those residing in rural areas with limited internet access. Consequently, our findings may not be generalized to these underrepresented groups, and prevalence estimates as well as associations should be interpreted with this caveat in mind. In future studies, stratified probability sampling or mixed-mode recruitment strategies should be employed to ensure broader population coverage. The RSES measures global rather than domain-specific self-esteem (e.g., body image, sexual self-esteem), which might show stronger associations. Our assessment of “conscious delay” relied on a single binary item that may not fully capture decision-making complexity.

Longitudinal designs with multiple postpartum assessments should be employed in future research in order to establish temporal precedence and examine bidirectional relationships. Experimental studies testing self-esteem interventions—body-positive peer groups, couples’ communication training, mindfulness-based self-compassion programs—would provide stronger causal evidence. Qualitative research exploring how women with varying self-esteem experience postpartum sexuality could provide richer mechanistic insights. Moderators (parity, birth mode, mental health history, cultural context) should be investigated in studies to identify the subgroups that would most benefit from self-esteem interventions. Given the significant role of partners (28, 37), dyadic studies examining both partners’ self-esteem and satisfaction would provide a more complete image. Intervention research should be performed to test whether self-esteem improvements translate into measurable sexual health and wellbeing benefits.

## 5 Conclusion

In this study, self-esteem is identified as a significant psychosocial resource for postpartum women, deeply interconnected with maternal competence, body image and partner relationship quality. Our findings demonstrate that higher self-esteem is associated with healthier patterns of sexual resumption, including less frequent conscious postponement and greater sexual activity frequency during the first postpartum year. These associations persist even when accounting for other relevant factors—positioning self-esteem as a key psychological correlate of postpartum sexual adjustment.

However, the decision to delay sexual resumption is multifactorial and associated not only with self-esteem, but also with birth experiences, previous sexual patterns and individual values related to self-respect. This complexity underscores the need for individualized, woman-centered approaches to postpartum sexual health support. These concerns are inadequately addressed in current healthcare practices, with most women reporting insufficient guidance from healthcare providers about sexual activity resumption.

Public health initiatives and clinical care models should shift beyond a purely biophysical approach to postpartum recovery and integrate strategies supporting and enhancing maternal self-esteem as a foundational element of holistic postpartum care. Routine postpartum visits should include non-judgmental discussions about body image, maternal confidence and sexual readiness. Support programs should actively work to validate diverse postpartum experiences, normalize gradual recovery timelines and celebrate maternal capabilities—all of which contribute to self-esteem. Healthcare providers, particularly midwives, require enhanced training in sexual health communication to effectively address these sensitive but crucial aspects of postpartum wellbeing.

By recognizing self-esteem as both an outcome worth supporting and a resource that facilitates other aspects of postpartum adjustment, we can develop more comprehensive, effective interventions promoting women’s overall health and quality of life during this transformative period.

## Data Availability

The original contributions presented in the study are included in the article/Supplementary material, further inquiries can be directed to the corresponding author.

## References

[1] Graziottin A Di Simone N Guarano A. Postpartum care: clinical considerations for improving genital and sexual health. Eur J Obstet Gynecol Reprod Biol. (2024) 296:250–7. doi: 10.1016/j.ejogrb.2024.02.037, 38484617

[2] Florkiewicz-Danel M Zaręba K Ciebiera M Jakiel G. Quality of life and sexual satisfaction in the early period of motherhood—a cross-sectional preliminary study. J Clin Med. (2023) 12:7597. doi: 10.3390/jcm12247597, 38137665 PMC10744264

[3] Behzadipour S Daneshpour M Damreihani N Aflatooni L. Sexual satisfaction and intimacy during pregnancy and after childbirth. Sexologies. (2021) 30:e111–7. doi: 10.1016/j.sexol.2020.10.002

[4] Belay HG Yehuala ED Asmer Getie S Ayele AD Yimer TS Berihun Erega B . Determinants of early resumption of postpartum sexual intercourse in sub-Saharan Africa: a systematic review and meta-analysis. Womens Health. (2024) 20:17455057241302303. doi: 10.1177/17455057241302303, 39610147 PMC11605757

[5] Artieta-Pinedo I Paz-Pascual C Garcia-Alvarez A Bully P Group ema Q Espinosa M. Resumption of sexual activity after childbirth and its related factors in Spanish women, a cross-sectional study. Midwifery. (2025) 141:104259. doi: 10.1016/j.midw.2024.104259, 39673987

[6] Dominoni M Gritti A Bergante C Pasquali MF Scatigno AL De Silvestri A . Genital perception and vulvar appearance after childbirth: a cohort analysis of genital body image and sexuality. Arch Gynecol Obstet. (2022) 307:813–9. doi: 10.1007/s00404-022-06826-4, 36309906 PMC9618153

[7] Rozmarynowska W Czerwińska-Osipiak A Krawczyk A Szablewska AW. The role of maternal age, education, birth experience and self-esteem in shaping anxiety before first postpartum intercourse. Front Public Health. (2025) 13:1708248. doi: 10.3389/fpubh.2025.1708248, 41458432 PMC12738329

[8] Fan X Zhou F Li Y Xia W Che Y. Factors associated with postpartum resumption of sexual intercourse among women in China: a retrospective multicenter study. J Obstet Gynaecol Res. (2022) 48:230–8. doi: 10.1111/jog.15088, 34788897

[9] Fernández-Fernández MJ de Medina-Moragas AJ. Comparative study of postpartum sexual function: second-degree tears versus episiotomy outcomes. Arch Gynecol Obstet. (2024) 309:2761–9. doi: 10.1007/s00404-024-07494-2, 38613578 PMC11147856

[10] O’Malley D Smith V Higgins A. Sexual health issues postpartum–a mixed methods study of women’s help-seeking behavior after the birth of their first baby. Midwifery. (2022) 104:103196. doi: 10.1016/j.midw.2021.103196, 34767981

[11] Ollivier R Aston M Price S Sheppard-LeMoine D Steenbeek A. ‘Feeling ready’: a feminist poststructural analysis of postpartum sexual health. Qual Health Res. (2024) 34:252–62. doi: 10.1177/10497323231209842, 37967315 PMC10768324

[12] Szablewska AW Michalik A Czerwińska-Osipiak A Zdończyk SA Śniadecki M Bukato K . Breastfeeding vs. formula feeding and maternal sexuality among polish women: a preliminary report. Healthcare. (2023) 12:38. doi: 10.3390/healthcare12010038, 38200943 PMC10779107

[13] Zamani M Moradi M Esmaily H Roudsari RL. The effectiveness of “women’s postpartum sexual health program (WPSHP)” on Iranian women’s postpartum sexual health: a randomized clinical trial. Health Care Women Int. (2024) 45:969–83. doi: 10.1080/07399332.2022.2083621 35857574

[14] Acele EÖ Karaçam Z. Sexual problems in women during the first postpartum year and related conditions. J Clin Nurs. (2012) 21:929–37. doi: 10.1111/j.1365-2702.2011.03882.x, 22008061

[15] Wassertheil S Cohen J. Statistical power analysis for the behavioral sciences. Biometrics. (1970) 26:588. doi: 10.2307/2529115

[16] Cuschieri S. The STROBE guidelines. Saudi J Anaesth. (2019) 13:S31–4. doi: 10.4103/sja.SJA_543_18, 30930717 PMC6398292

[17] Łaguna M Lachowicz-Tabaczek K Dzwonkowska I. Skala samooceny SES Morrisa Rosenberga – polska adaptacja metody. Psychologia Społeczna. (2007) 2:164–76.

[18] World Health Organization. Sexual Health and its Linkages to Reproductive Health: an Operational Approach. Geneva: World Health Organization (2017).

[19] van Scheppingen MA JaapJA D Chung JM Tambs K Bleidorn W. Self-esteem and relationship satisfaction during the transition to motherhood. J Pers Soc Psychol. (2018) 114:973–91. doi: 10.1037/pspp0000156, 28795822

[20] Hutchinson J Cassidy T. Well-being, self-esteem and body satisfaction in new mothers. J Reprod Infant Psychol. (2022) 40:532–46. doi: 10.1080/02646838.2021.1916452, 33877938

[21] Bdier D Mahamid F Fallon V Amir M. Posttraumatic stress symptoms and postpartum anxiety among palestinian women: the mediating roles of self-esteem and social support. BMC Womens Health. (2023) 23:420. doi: 10.1186/s12905-023-02567-x, 37559047 PMC10413689

[22] Meireles JFF Neves CM Amaral ACS da R MFF MEC F Appreciation B. Depressive symptoms, and self-esteem in pregnant and postpartum Brazilian women. Front Glob Womens Health. (2022) 3:834040. doi: 10.3389/fgwh.2022.834040, 35368995 PMC8970598

[23] Zayed AA El-Hadidy MA. Sexual satisfaction and self-esteem in women with primary infertility. Middle East Fertil Soc J. (2020) 25:13. doi: 10.1186/s43043-020-00024-5

[24] Martínez-Martínez A Arnau J Salmerón JA Velandrino AP Martínez ME. The sexual function of women during puerperium: a qualitative study. Sex Relatsh Ther. (2017) 32:181–94. doi: 10.1080/14681994.2016.1263389

[25] DeMaria AL Delay C Sundstrom B Wakefield AL Avina A Meier S. Understanding women’s postpartum sexual experiences. Cult Health Sex. (2019) 21:1162–76. doi: 10.1080/13691058.2018.1543802 30624136

[26] Wallwiener S Müller M Doster A Kuon RJ Plewniok K Feller S . Sexual activity and sexual dysfunction of women in the perinatal period: a longitudinal study. Arch Gynecol Obstet. (2017) 295:873–83. doi: 10.1007/s00404-017-4305-0 28251311

[27] Sinclair SJ Blais MA Gansler DA Sandberg E Bistis K LoCicero A. Psychometric properties of the Rosenberg self-esteem scale: overall and across demographic groups living within the United States. Eval Health Prof. (2010) 33:56–80. doi: 10.1177/0163278709356187 20164106

[28] Li L Xiao L Yuan X Wu J Li J Chen X . Partner involvement and emotional and informational support gaps as predictors of postpartum birth trauma symptoms: a multi-center cross-sectional study of 230 women at 42 days postpartum. BMC Pregnancy Childbirth. (2025) 25:918. doi: 10.1186/s12884-025-08084-z 40898125 PMC12403439

[29] Fodstad K Staff AC Laine K. Sexual activity and dyspareunia the first year postpartum in relation to degree of perineal trauma. Int Urogynecol J. (2016) 27:1513–23. doi: 10.1007/s00192-016-3015-7 27185318

[30] Byers-Heinlein A McCallum A Byers ES Pukall C. Sexual health-related training of Canadian midwives and its association with practice outcome. Women Birth. (2020) 33:e199–207. doi: 10.1016/j.wombi.2019.03.017 31003937

[31] Darooneh T Ozgoli G Keshavarz Z Nasiri M Khiabani A Roosta F. Development of an educational guide for postpartum sexual health promotion: the Delphi method. J Educ Health Promot. (2023) 12:444. doi: 10.4103/jehp.jehp_1839_22 38464651 PMC10920744

[32] Knutson AJ Boyd SS Long JB Kjerulff KH. Early resumption of sexual intercourse after first childbirth and unintended pregnancy within six months. Womens Health Issues. (2022) 32:51–6. doi: 10.1016/j.whi.2021.09.002 34602327

[33] Kul Uçtu A Karakoç H. Breastfeeding sexual lives of first-time mothers: a qualitative explorative study. Breastfeed Med. (2022) 17:1025–33. doi: 10.1089/bfm.2022.0194 36475936

[34] Alp Yllmaz F Sener Taplak A Polat S. Breastfeeding and sexual activity and sexual quality in postpartum women. Breastfeed Med. (2019) 14:587–91. doi: 10.1089/bfm.2018.0249 31298557

[35] Zgliczynska M Zasztowt-Sternicka M Kosinska-Kaczynska K Szymusik I Pazdzior D Durmaj A . Impact of childbirth on women’s sexuality in the first year after the delivery. J Obstet Gynaecol Res. (2021) 47:882–92. doi: 10.1111/jog.14583 33372310

[36] Główny Urząd Statystyczny (Central Statistical Office). "Narodowy Spis Powszechny Ludności i Mieszkań 2021 Ludność. Stan i struktura demograficzno-społeczna w świetle wyników NSP 2021". In: National Population and Housing Census 2021 Population. Size and Demographic-social Structure in the light of the 2021 Census Results. Warsaw: (2023)

[37] Tavares D Fidalgo D Sousa M Morais A Jongenelen I Lamela D . History of mental health problems moderates the association between partner support during childbirth and women’s mental health in the postpartum period. Midwifery. (2025) 144:104359. doi: 10.1016/j.midw.2025.104359 40049016

