# Supplementary material for: Maternal self-esteem and postpartum sexual health in Polish women
Source: Front Public Health. 2026 Jun 16;14:1813933. doi: 10.3389/fpubh.2026.1813933 (PMC13314957; doi:10.3389/fpubh.2026.1813933)
Supplement: Supplementary file 1 [file Table_1.DOCX]

Supplementary table S1. Strong and moderate correlates of self-esteem in postpartum period

| **Self-esteem SES interpretation** | | | | |
| --- | --- | --- | --- | --- |
| **Correlates** | **Low** | **Moderate** | **High** | **Test results** |
| STRONG CORRELATES | | | | |
| Childcare competence | | | | χ²(4): 35.69, *p* < 0.001 |
| Bad | 50,00% | 33,33% | 16,67% |  |
| Good | 25,12% | 47,29% | 27,59% |  |
| Very good | 14,89% | 26,95% | 58,16% |  |
| Sexual attractiveness | | | | χ²(4) = 51.36, *p* < 0.001 |
| Cannot say | 19.39% | 46.94% | 33.67% |  |
| No | 36.50% | 37.23% | 26.28% |  |
| Yes | 5.22% | 33.91% | 60.87% |  |
| Partner closeness | | | | χ²(2) = 23.48, *p* < 0.001 |
| No | 42.31% | 44.23% | 13.46% |  |
| Yes | 17.79% | 37.92% | 44.30% |  |
| MODERATE CORRELATES | | | | |
| Age | | | | χ²(6) = 14.78, *p* = 0.022 |
| 24 years and below | 26.32% | 43.86% | 29.82% |  |
| 25-29 years | 22.09% | 42.44% | 35.47% |  |
| 30-34 years | 17.82% | 35.64% | 46.53% |  |
| 35 years and above | 20.00% | 10.00% | 70.00% |  |
| Breastfeeding | | | | χ²(2) = 6.25, *p* = 0.044 |
| No | 46.67% | 51.47% | 36.69% |  |
| Yes | 53.33% | 48.53% | 63.31% |  |
